# Active feature elicitation: An unified framework

**DOI:** 10.3389/frai.2023.1029943

**Published:** 2023-03-24

**Authors:** Srijita Das, Nandini Ramanan, Gautam Kunapuli, Predrag Radivojac, Sriraam Natarajan

**Affiliations:** ^1^Department of Computing Science, University of Alberta, Edmonton, AB, Canada; ^2^Palo Alto Networks, Santa Clara, CA, United States; ^3^Computer Science Department, University of Texas at Dallas, Dallas, TX, United States; ^4^Khoury College of Computer Sciences, Northeastern University, Boston, MA, United States; ^5^Computer Science Department, University of Texas at Dallas, Dallas, TX, United States

**Keywords:** active learning, feature elicitation, classification, healthcare, sample-efficiency

## Abstract

We consider the problem of active feature elicitation in which, given some examples with all the features (say, the full Electronic Health Record), and many examples with some of the features (say, demographics), the goal is to identify the set of examples on which more information (say, lab tests) need to be collected. The observation is that some set of features may be more expensive, personal or cumbersome to collect. We propose a classifier-independent, similarity metric-independent, general active learning approach which identifies examples that are dissimilar to the ones with the full set of data and acquire the complete set of features for these examples. Motivated by four real clinical tasks, our extensive evaluation demonstrates the effectiveness of this approach. To demonstrate the generalization capabilities of the proposed approach, we consider different divergence metrics and classifiers and present consistent results across the domains.

## 1. Introduction

Acquiring meaningful data is important for learning robust models, and is especially relevant in data scarce domains such as medicine. While there are a plethora of data regarding several diseases, in some cases, it is crucial to obtain information that is particularly relevant to the learning task. The problem of choosing an example to obtain its class label has been addressed as active learning (Settles, 2012). There have been several extensions of active learning that included presenting a set of features (Raghavan et al., 2006; Druck et al., 2009), or getting labels over clusters (Hofmann and Buhmann, 1998), or preferences (Odom and Natarajan, 2016) or in sequential decision making (Lopes et al., 2009), to name a few. Most of these directions considered getting label information for a set of unlabeled examples.

**Figure 1:**
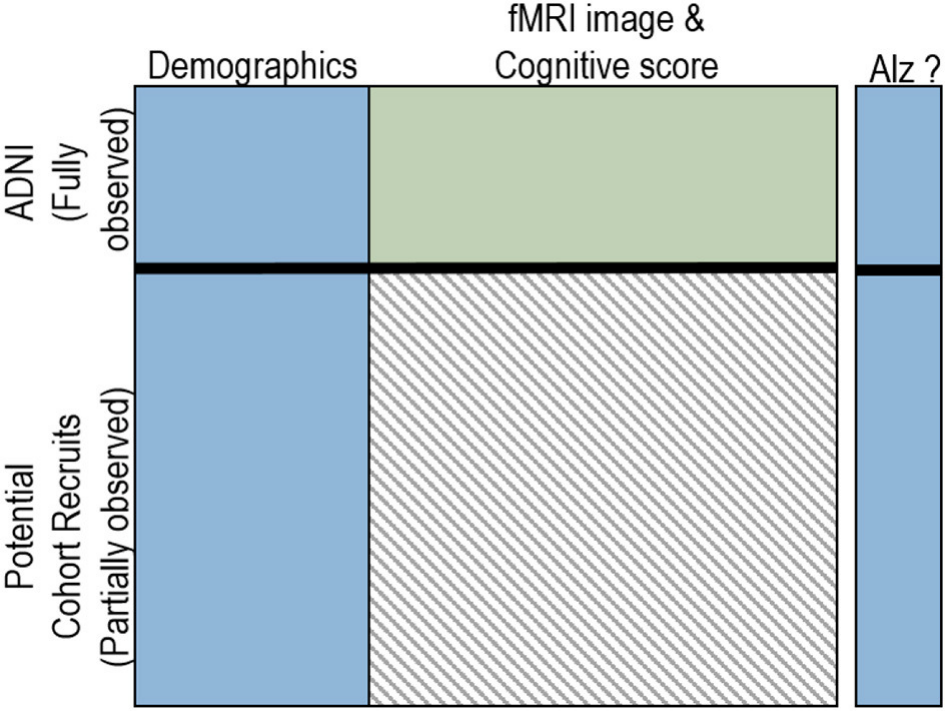
The active feature elicitation setting. The top part is the fully observed data and the bottom right (gray shaded area) is the elicitable feature set. Figure appears in Natarajan et al. (2018).

Our problem is different and is motivated by a set of medical tasks with a common requirement—that of recruiting patients for a clinical study. Consider the following scenario of collecting data (cognitive score and fMRI, both structural and functional) for an Alzheimer's study. Given a potentially large cohort size, the first step could be to simply collect the demographic information on everyone. Now, given a small amount of complete data from a related study, say the Alzheimer's Disease Neuro-Initiative (ADNI), our goal is to recruit subjects who would provide the most information for learning a robust, generalized model. This scenario is highlighted in Figure 1. The top part shows the part of the data that is fully observed (potentially from a related study). The bottom left quadrant shows the observed features of the potential cohorts and the right quadrant is the data that needs to be collected for the most useful potential recruits (we refer to these features as *elicitable features* from hereon). Given, the labels of the potential recruits, the goal is to identify the most informative cohorts that would aid the study. The definition of *most informative* is quite general in our work and can be adapted as necessary.

Inspired by the success of active learning methods, we define the problem of *active feature elicitation* (AFE) where the goal is to select the best set of examples on whom the elicitable features can be queried on to best improve the classifier performance. At a high level, our algorithm (also called AFE) at each iteration, identifies examples that are most different from the current set of fully observed examples. These are then queried for the elicitable features, their feature-values are obtained and added to the training set. Then, the models are updated and the process is repeated until convergence. This is a *general-purpose* framework. Any distance metric that works well with the data and the model can be employed. So can a classifier that is capable of handling the specific intricacies of the data. Finally, the convergence criteria can be decided based on the domain. We provide a list of such distances and criteria after introducing the algorithm.

### 1.1. Motivating medical tasks

We motivate active feature elicitation with four medical tasks that we address in this work:

1. **Parkinson's**: The Parkinson's Progression Markers Initiative (PPMI) is an observational study with the aim of identifying biomarkers that impact Parkinson's progression (Marek and Jennings, 2011). The data can be divided broadly into four categories: imaging data, clinical data, bio-specimens and demographics. Among these data types, while other modalities are either costly or cumbersome to obtain, the total Montreal Cognitive Assessment Score (MoCA) is a standard measure that can be used to select subjects for whom information from other modalities can improve classifier performance significantly. Given that the other metrics are expensive/cumbersome to collect, one could potentially only employ the demographic information and query the most informative subjects to collect information of the other modalities.
2. **Alzheimer's**: The Alzheimer's Disease NeuroIntiative (ADNI^1^) aims to test whether serial MRI, PET, biological markers, and clinical and neuropsychological assessments can measure the progression of mild cognitive impairment and early Alzheimer's disease. Given a small number of subjects with all the measurements, demographic data can be used to select subjects for whom obtaining more information such as the cognitive MMScore and imaging data could maximize performance in predicting Alzheimer's progression. Specifically, one could determine the set of subjects on whom the expensive imaging tests could be performed based on the demographic and questionnaire information.
3. **Rare diseases**: A recent work (MacLeod et al., 2016) focused on predicting rare diseases from a survey questionnaire that consisted of questions in the following categories: demographics, technology use, disease information and healthcare provider information. The set of diseases in the study includes Ehlers Danlos Syndrome (23%), Wilson's Disease (21.9%), Kallmann's Syndrome (9.9%), etc. Demographics can be used to identify the future participants of the survey as this can avoid more personal questions such as technology use and the provider details along with the disease information itself.
4. **Post-partum depression**: This work collects demographic information along with several sensitive questions including relationship troubles, social support, economic status, infant behavior and the CDC questions to identify PPD in subjects outside the clinic (Natarajan et al., 2017). As with the earlier cases, demographics can be used to recruit the subjects on whom more sensitive information can be collected.

These varied medical tasks demonstrate the need for employing an active feature elicitation approach that allows for collecting relevant information in an effective manner. While the presented motivating tasks are medical, one could imagine the use of such an approach in any domain where some features are either expensive or cumbersome to obtain. While our work has been motivated as improving a specific performance metric such as accuracy, the framework is general enough to incorporate any metric including more recently addressed *fairness metrics* (Dwork et al., 2012; Kleinberg et al., 2017; Gillen et al., 2018).

**Contributions:** We make a few key contributions. First, we identify and formally define the problem of actively acquiring features from a selected set of examples. Second, we show the potential of this approach in four real medical prediction tasks: Alzheimer's from fMRI and cognitive score, Parkinson's from potential risk factors, rare diseases based on a survey questionnaire and predicting post-partum depression (PPD) in a non-clinical setting. Third, we present empirical evidence based on different divergence measures and different learning algorithms to validate the generality of the proposed approach. Finally, we empirically demonstrate that AFE is particularly effective in recall while not sacrificing the performance in these four real tasks.^2^

The rest of the paper is organized as follows: first, we introduce the necessary background and discuss the related work. Then, we introduce our framework of AFE and present the different divergence measures that can be expanded. We next present the experimental domains and perform a comprehensive analysis across different divergence measures and base classifiers. We then conclude the paper by presenting interesting directions for future research.

## 2. Background and related work

Our work is closely related to active learning where the most informative examples are chosen based on a scoring metric to be labeled when learning with semi-supervised data. Active learning (Settles, 2012) relies on the fact that if an algorithm can only solicit labels of a limited number of examples, then it should choose them judiciously since not all examples provide the same amount of information. Active learning has a long history of being successfully employed with a variety of classifiers such as logistic regression (Lewis and Catlett, 1994; Lewis and Gale, 1994), support vector machines (Tong and Koller, 2001b), Bayesian network learning (Tong and Koller, 2000, 2001a) and in sequential decision making tasks such as imitation learning (Judah et al., 2014) and inverse reinforcement learning (Odom and Natarajan, 2016).

Active learning has also been used with elicitable features. Zheng and Padmanabhan (2002), for instance, considered a setting where an imputation method was used to fill the incomplete feature subset and used scoring methods to acquire the most informative example for labeling. While their method explicitly estimated the missing feature subset using an imputation model for each instance to compute the score, our algorithm is not dependent on any imputation model and does not use any estimate of the feature subset to be acquired. Melville et al. (2004) used uncertainty sampling to acquire the maximally informative unlabeled examples from the partially observed set of features during training time. Their model to compute uncertainty also depends on some predefined imputation strategy. While Zheng et al. uses the examples with the complete feature set to build their model and Melville et al. uses all of the available training examples to build their model, our method uses two different models to capture uncertainty. There is also a different body of work where instances are chosen based on individual feature utilities. Melville et al. (2005) computed expected feature utility for every feature per example based on how acquiring each feature will have an impact on the model's performance per unit cost while (Lizotte et al., 2003) pose the problem as Sequential decision making task and uses expected feature utility to learn the desired policy while fixing their budget a priori. While our work is significantly different in motivation and technicality to the above-mentioned literature, all of these methods are somewhat similar to our work in the problem setting of identifying the best example or best feature per example to acquire during training time.

Our work is inspired by the work of Kanani and Melville (2008) on *active feature acquisition* that addressed a similar problem where a few examples with the full set of features are present while others are incomplete examples. Their work also scored these examples based on uncertainty sampling and then updated the model at prediction time. There are a few key differences between this work and ours. First, our model updates occur *during training* and *not during the test time*. Second, we return a single model on the best set of training data while their approach had two different models for testing. Our approach explicitly grows the set of training examples for a single model iteratively. They employed uncertainty sampling on the observed feature sets (which is a baseline in our approach), while we explicitly compute the distance between the two distributions induced by our model. Finally, while their test data can have partial feature sets, our method evaluates test time performance on instances with the complete feature set available. Kanani et al. (2007) also looked at the test time acquisition problem for related entities to do entity resolution.

This work was later extended by Thahir et al. (2012) for protein-protein interaction prediction where an extra term was added to the utility function that explicitly computed the value of adding an example to the classifier set. While it is possible to compute the value of adding an example to the training set in our work, we will pursue this as a future research direction. The AFA framework was then later generalized and rigorously analyzed by Saar-Tsechansky et al. (2009) where even class labels can be considered to be missing and acquired. We assume that these labels are observed and that full sets of features need to be acquired. A key difference to this general direction is that both the observed set of examples and observed set of features are significantly smaller in our work compared to the general AFA setting which is clearly demonstrated in our experiments.

Bilgic and Getoor (2007) took a different approach to a similar task where they assumed different costs for misclassification and information acquisition. They proposed a probabilistic framework that explicitly modeled this dependency and developed an algorithm to identify the set of features that can be optimally identified. Using such a strategy for discovering sets of features that one could acquire for different sets of patients is an interesting direction. Feature elicitation is inspired by the preference framework of concept learning by Boutilier et al. (2009), where minimax regret is used for computing the utility of subjective features. The violated constraints are repeatedly added to the computation and can potentially make the problem harder to solve. Huang et al. (2018) solved a different variant of the problem where different features are missing for different examples at training time and they used matrix completion with active learning scoring strategies to acquire the most informative features for various examples. While their problem setting assumes different parts of the feature vector to be missing, our problem setting assumes a fixed part of the feature vector to be missing during training. A variant of this problem was posed by Krause and Guestrin (2009) as subset-selection problem solved by optimizing the feature-level value of information from graphical models.

There is another line of work that uses sequential decision making to learn an optimal policy to decide which features and in what order needs to be acquired. As discussed earlier, Lizotte et al. (2003) pose the problem of acquiring features as a sequential decision-making problem in a budgeted setting and learn the desired policy. A few directions of work (Dulac-Arnold et al., 2011; Shim et al., 2018) pose the problem of acquiring the best feature subset for every example at prediction time as a joint optimization problem where the learning agent optimizes the acquisition cost for every feature using a reward function which is the expected classifier performance. The work by Dulac-Arnold et al. (2011) was extended by Janisch et al. (2019) who replaced the linear components with neural networks and used Deep Q-networks to estimate the utility of each feature. He et al. (2012) used imitation learning to solve the problem of feature selection by demonstrating immediate best actions (may not be the best long term actions) for the learner, thus providing the learner with trajectories demonstrating sub-optimal policy. While all of the above-mentioned work deals with the problem of acquiring the most informative feature subsets for different examples at prediction time, our method focuses on deciding the most informative example for which a fixed predefined feature subset needs to be acquired at training time. Similar to these methods, our method can also accommodate cost-sensitive learning by including a cost-sensitive classifier (Chai et al., 2004; Ling et al., 2004) to calculate the divergence measure.

A slightly different but related direction is prediction time feature elicitation. Nan et al. (2015, 2016) incorporated a budget constraint along with the loss function of random forests to trade off between acquisition cost and accuracy. Other tree based models including gradient boosted trees and directed acyclic graphs was used by Xu et al. (2012), Xu et al. (2013), and Wang et al. (2015) to optimize test-time acquisition cost. A subsequent work (Nan and Saligrama, 2017) adaptively used a low prediction cost model wherever possible and switched to a high cost model in difficult regions of input space. Gong et al. (2019) used latent gaussian models to address the problem of acquiring features at training time and also applied it to a real world health care data. A recent work by Das et al. (2021) identified important feature subsets for different examples during training by optimizing an information-theoretic feature-selector function thus helping in cost effective feature elicitation during testing. In all of the aforementioned work, a subset of features is elicited at prediction/training time. In our problem setting, examples are selected actively to build a training model with most informative training data and the entire feature set is elicited for the chosen examples.

To summarize, our work is inspired by the contributions from several of these related works but **differs** in the motivation of collecting more features by identifying the right set of examples during training time to improve the model. As mentioned, we differ from active feature acquisition in both motivation and execution—we collect a large number of features from a small set of examples during training and use distances to calculate the most diverse set of examples.^3^ The other important difference is in the number of observed features, which is assumed to be much smaller in our work. And our solution that explicitly computes the relationship between the observed and unobserved data is independent of the choices of classifiers and distance functions. One of the key assumptions that we make is that all elicitable features are collected for the selected examples, and identifying the relevant set of features along the lines of Bilgic and Getoor is an exciting direction for future work.

## 3. Active feature elicitation

Let us denote the label of an example *i* as *Y*^*i*^, the set of fully observed features (i.e., the features that are observed for the entire data set) as **X**_*o*_, the set of elicitable features as **X**_*u*_, the set of fully observed examples set as ${\text{E}}_{o}=\langle \langle {\text{X}}_{o}^{1},{\text{X}}_{u}^{1},Y^{1}\rangle ...\langle {\text{X}}_{o}^{k},{\text{X}}_{u}^{k},Y^{k}\rangle \rangle$ and the set of partially observed examples as ${\text{E}}_{u}=\langle \langle {\text{X}}_{o}^{1},Y^{1}\rangle ...\langle {\text{X}}_{o}^{\ell },Y^{\ell }\rangle \rangle$. The learning problem in our setting can be defined as follows:

**Given:** A data set with **E**_*o*_ and **E**_*u*_.

**To Do:** Identify the best set of examples **E**_*a*_ ⊂ **E**_*u*_ for which to obtain more information **X**_*u*_ such that the classifier performance improves.

In the above definitions, the notion of *best* and *improve* have been *intentionally* left vague. This definition allows for any notion of the best examples and improvement of the classifier. In our work, to be precise, we consider *best* to denote the set of the examples with maximal divergence to the observed set and *performance* to be the log-likelihood of the classifier. It is to be noted that the proposed divergence-based example selection strategy is not necessarily the best but is presumably better than other existing alternative active-learning strategies like uncertainty sampling as observed empirically. The classifier we consider is the well-understood gradient-boosting (Friedman, 2001) and Support Vector Machines (Vapnik, 2013).

Since our focus is on clinical (study) data, our hypotheses is that the best examples are chosen to obtain extra information from those that are significantly different from the remaining examples. In principle, any distance function could be used to determine the set of examples **E**_*a*_ from **E**_*u*_ that are significantly different from the ones in **E**_*o*_. We use the *mean divergence* between every example $e_{i}=\langle {\text{X}}_{o}^{i},Y^{i}\rangle$ in **E**_*u*_ and every example $e_{j}=\langle {\text{X}}_{o}^{j},{\text{X}}_{u}^{j},Y^{j}\rangle$ in **E**_*o*_ to determine the set of examples in **E**_*u*_ that are different from the observed set **E**_*o*_. To compute this mean divergence at every iteration *t*, we use the current models: $M_{u}^{t}=P_{t}\left(Y^{i}|{\text{X}}_{o}^{i}\right)$ and $M_{o}^{t}=P_{t}\left(Y^{j}|{\text{X}}_{o}^{j},{\text{X}}_{u}^{j}\right)$, learned on the two different sets of data. More precisely, we compute the mean distance of an example ${\text{X}}_{u}^{i}$ from all the observed examples $\langle {\text{X}}_{o}^{j},{\text{X}}_{u}^{j}\rangle$ as,


(1) $$\begin{array}{l} {MD}_{i}=\frac{1}{\left|{\text{E}}_{o}\right|}\overset{\left|{\text{E}}_{o}\right|}{\underset{j=1}{\sum }}D_{ij}, \end{array}$$

where the distance *D*_*ij*_ can be asymmetric KL divergence, Hellinger distance, χ^2^-distance or Total-variation:


(2) $$\begin{array}{l} D_{ij}=Div\left(P\left(Y^{i}|{\text{X}}_{o}^{i}\right)∥P\left(Y^{j}|{\text{X}}_{o}^{j},{\text{X}}_{u}^{j}\right)\right). \end{array}$$

A natural question to ask is: what is the need for two different distributions, even if they are conditionals on the target. Note that, with the set of examples **E**_*o*_, all the features are assumed to be fully observed. Ignoring the informative features (${\text{X}}_{u}^{j}$) when computing the distances can lead to a loss of information and our experiments confirmed this. Hence, we employ the model learned over the full set of features for the fully observed example set **E**_*o*_, which is typically smaller than the elicitable set in the initial iterations.

Now that the distances have been computed, we next sort them to pick the *n* most distinct examples from **E**_*u*_. These *n* examples are queried for their elicitable features and are then added to the training set before the model is retrained. Note that at each iteration, the model $P\left(Y^{j}|{\text{X}}_{o}^{j},{\text{X}}_{u}^{j}\right)$ is updated after the examples are appropriately chosen and queried. The model $P\left(Y^{i}|{\text{X}}_{o}^{i}\right)$ remains unchanged because it is trained on **X**_*o*_ of the entire example set **E**_*o*_ ∪ **E**_*u*_. The process is repeated until convergence or a predetermined budget is realized. Although the final goal of our problem is to maximize the log-likelihood of observed training data for achieving better test-time performance, it is not possible to do so without an example selection strategy because we assume that elicitable features needs to be acquired at a cost and the entire training data is not observed prior to training. The different divergence metric helps to identify the data-points that are most dissimilar to the observed data distribution, hence augments the observed training data with examples that provide maximum information to the model for optimizing the underlying objective (maximizing log-likelihood in this problem). This helps the model to achieve good performance while balancing the cost of feature acquisition.

We present a generalized and unifying framework, which can be adapted in multiple ways:

1. As we discuss in Section 3.1, this formulation admits a large class of divergences and distance metrics for computing distances between examples in **E**_*o*_ and **E**_*u*_. To demonstrate this generality, we considered several different measures—*KL-divergence, Hellinger distance* χ^2^ *distance* and *Total variation* distances. One could imagine the use of other classifiers, kernels or learned metrics (Kunapuli and Shavlik, 2012) as well.
2. The gradient boosting classifier can be replaced with any classifier. An appropriate choice of divergence can greatly benefit from the choice of the classifier. Our framework is classifier-agnostic, allowing the user to select the best one for the task at hand. To demonstrate this, we have considered SVMs as the base classifier as well.
3. Various convergence criteria can also be used. For instance, one could simply preset the number of iterations, or employ a tuning set to determine the change in performance from the previous iteration or compute the difference between scores from successive iterations. We employ this final strategy: computing the difference between log-likelihoods of the training data in successive iterations. If the difference is smaller than ϵ, we terminate the algorithm. One could also imagine reducing the number of queries at every iteration (i.e., successively reduce $n=\frac{n}{n+\Delta }$) such that the number of examples selected at each iteration naturally comes down to 0.

We present the algorithm (Natarajan et al., 2018) for active feature elicitation in Algorithm 1. The AFE algorithm takes as input the set of fully labeled examples (**E**_*o*_), the set of partially labeled examples (**E**_*u*_), the number of active learning examples for each query step (*n*) and step size (Δ). In *this algorithm*, sufficient decrease in step size ($\frac{n}{n+\delta }$) is used as the stoppage criterion (lines 4, 21) as an example. This can be replaced by other task-relevant budgets or convergence criteria, as discussed previously.

**Algorithm 1:** Active Feature Elicitation.

1: function ActiveFeatureElicitation(**E**_*o*_, **E**_*u*_, *n*, Δ)
2: *t* = 0 ⊳ iteration counter
3: *M*_*t*_= TrainInitialModel(**E**_*o*_, **E**_*u*_, **X**_*o*_, **X**_*u*_)
4: while *n*≥1 **do** ⊳ while not converged
5: MD = **0** ⊳ initialize mean divergences
6: for *i* = 1 **to** |**E**_*u*_| **do**
7: **D** = **0** ⊳ init divergence for unobserved ex. *i*
8: for *j* = 1 **to** |**E**_*o*_| **do**
9: *D*_*j*_= ComputeDistance(*E*_*i*_, *E*_*j*_, *M*_*t*_)
10: end **for**
11: MD_*i*_ = $\frac{\overset{\left|{\text{E}}_{o}\right|}{\underset{j=1}{\sum }}D_{j}}{\left|{\text{E}}_{o}\right|}$ ⊳ average distance
12: end **for**
13: ${\text{E}}_{u}^{q}=$ GetTopN(MD)
14: ⊳ *n* most divergent partially-observed examples
15: ${\text{E}}_{o}^{q}=$ AppendNewFeature$\left({\text{E}}_{u}^{q}\right)$
16: ⊳ actively query to obtain elicitable features
17: **E**_*o*_ = ${\text{E}}_{o}\cup {\text{E}}_{o}^{q}$ ⊳ add queried to observed
18: **E**_*u*_ = ${\text{E}}_{u}\backslash {\text{E}}_{u}^{q}$ ⊳ remove queried from unobs.
19: *M*_*t*_= UpdateModel(**E**_*o*_, **E**_*u*_, **X**_*o*_, **X**_*u*_)
20: ⊳ retrain or update classifier
21: $n=\frac{n}{n+\Delta }$ ⊳ check convergence/update budget
22: end **while return** TrainFinalModel(**E**_*o*_)
23: end **function**

After initializing mean distances of each unlabeled example, AFE iterates through every partially-labeled example in **E**_*u*_, and computes the mean distance to all the fully labeled examples in **E**_*o*_ based on the divergence between the respective current models. The *n*-most divergent (dissimilar) examples are selected and features are actively obtained for these examples (AppendNewFeature). These examples are then added to **E**_*o*_ and removed from **E**_*u*_. A new model can be trained (or updated, depending on the choice of classifier), and the process is repeated. Note that *M*_*t*_ consists of two classifiers—one trained on **E**_*o*_, which contains new examples provided by the user after active feature elicitation with all the features, and the other trained on entire data **E**_*o*_ ∪ **E**_*u*_. After convergence, **E**_*o*_ has the full set of training examples.

As mentioned earlier, one could employ any distance (or pseudo distance) metrics, any compatible classifier and multiple convergence criterion. In our experiments, we employ both Gradient boosting and Support Vector Machines as the classifier; KL-divergence, Hellinger distance, χ^2^-distance and Total-variation as the distance measures to identify the unobserved examples whose features we would like to elicit, and the difference in average log-likelihoods between two iterations as our convergence criterion.

### 3.1. Other model divergences

The KL divergence is a special case of the Csiszár *f*-divergence (Csiszár, 1967), which is a *generalized measure* for the difference between two probability distributions, in our case $P\left(Y^{i}|{\text{X}}_{o}^{i}\right)$ and $P\left(Y^{j}|{\text{X}}_{o}^{j},{\text{X}}_{u}^{j}\right)$. $D_{f}\left(P∥Q\right)=\int_{\Omega }f\left(dP/dQ\right)dQ$.

Generally, given two distributions *P* and *Q* over some space Ω, for a convex function *f* (with *f* (1) = 0), the divergence of *P* from *Q* is defined as


(3) $$\begin{array}{l} D_{f}\left(P∥Q\right)=\int_{\Omega }f\left(dP/dQ\right)dQ \end{array}$$

Such *f*-divergences satisfy non-negativity, monotonicity, and convexity, though they are *not always symmetric*. Several well-known distribution distance measures are special cases of the *f*-divergence and are shown in Table 1 and are used in our experiments for evaluation. For example, the χ^2^-divergence might be well-suited for histogram data (Kedem et al., 2012), while the Hellinger distance might benefit applications with highly-skewed data (Cieslak et al., 2012).

**Table 1:** Several well-known *f*-divergences for discrete distributions (where **p** and **q** are vector representations of the distributions) are shown.

**Divergence**	***f*(*x*)**	***D*_*f*_(p∥q)**
KL-divergence	*x*log*x*	$\underset{i}{\sum }p_{i}\log\frac{p_{i}}{q_{i}}$
Hellinger distance	${\left(\sqrt{x}-1\right)}^{2}$	$\frac{1}{\sqrt{2}}||\sqrt{\text{p}}-\sqrt{\text{q}}|{|}_{2}$
Total variation	$\frac{1}{2}|x-1|$	$\frac{1}{2}||\text{p}-\text{q}|{|}_{1}$
Neyman χ^2^-divergence	(*x*−1)^2^	$\underset{i}{\sum }\frac{{\left(p_{i}-q_{i}\right)}^{2}}{q_{i}}$

Their continuous extensions can be obtained by replacing the sums with integrals over the support of the distributions.

Recently it was shown that families of divergences including the *α*- and *β*-divergence are also special cases of the *f*-divergence (Cichocki and Amari, 2010). The latter includes generalizations of measures such as the Euclidean distance and the Itakura-Saito distance, which are appropriate for unsupervised and semi-supervised learning problems.^4^ We consider the usefulness of various divergences to different machine learning problem types and applications in future work.

### 3.2. Multi-class and other extensions

As our approach is algorithm- and divergence-agnostic, it can be seamlessly extended to multi-class settings. As long as the underlying classification algorithm can produce (multinomial) distributions over the label space: $\text{p}=P\left(Y^{i}|{\text{X}}_{o}^{i}\right)$ and $\text{q}=P\left(Y^{j}|{\text{X}}_{o}^{j},{\text{X}}_{u}^{j}\right)$, we can use any model divergence discussed in Section 3.1.

There are several other possible extensions of the proposed approach. First is the necessity to move beyond active learning; while standard methods acquire a label for each example, in many situations where the goal is to understand why an event happens (such as clinical studies), it is necessary to obtain more tests/features. Also, given that the original model is learned from a small set of features, the model will not be necessarily generalizable. Second, it is possible that some specific set of features are the most informative for a specific example. For instance, some subjects' predictions will benefit from some lab test while a different test is a better indicator for someone else. Extending our framework to handle these different types of examples/feature combinations is outside the scope and is an interesting future direction.

## 4. Empirical evaluation

We now present evaluation results on one standard UCI data set (PIMA, Smith et al., 1988) and *four real medical tasks* to demonstrate the efficacy of our approach. It must be mentioned clearly that while healthcare is one domain, the data sets are varied: from online behavior to images to risk factors to survey. The goal is to demonstrate the versatility of the approach with real problems and also that this is a unified framework whose components can be plugged in according to the domain, data and prior knowledge. It must also be noted that while healthcare is considered in this work, the ideas are not limited to this domain and any problem where a small set of data is fully observed and the rest are partially observed can render itself as a useful domain for the proposed approach.

1. **Parkinson's prediction from clinical study:** The task is to predict the occurrence of Parkinson's disease from different modalities. We focus on a smaller set of features, primarily motor and non-motor assessments resulting in a set of 37 attributes including the class label. The observed feature is the MoCA test result, while the other 35 motor scores are treated as elicitable.
2. **Alzheimer's prediction from ADNI:** We assume that demographics are observed, while cognitive score (MMScore) and fMRI image features are elicitable. We use the AAL Atlas^5^^,^^6^ to segment the image into 108 Regions of Interest (RoIs), and for each RoI, we derive their summary attributes: white matter, cerebral spinal fluid, and gray matter intensities along with regional variance, size and spread.
  While the original data set has three classes: Alzheimer's (AD), Cognitively Normal (CN), and Mildly Cognitively Impaired (MCI), we consider the binary task of predicting AD vs. the rest. The presence of MCI subjects makes this particular task challenging, yet interesting; this is because these subjects may or may not end up having Alzheimer's eventually. Identifying the right set of subjects to target for feature elicitation can considerably improve classifier performance, as we show below.
3. **Rare disease prediction from self-reported survey data:** The task is to predict if a subject has a rare disease (MacLeod et al., 2016); by definition, a rare disease is hard to diagnose and affects less than 10% of the world's population. The data for this prediction task arises from survey questionnaires and we assume that demographic data are fully observed. Other survey answers concerning technology use, disease information and healthcare details are treated as elicitable.
4. **Post-partum depression prediction from online questionnaire data:** Recently, Natarajan et al. (2017) employed online questionnaires to predict PPD from demographics, social support, relevant medical history, childbirth issues, and screening data. We assume that demographics are observed and are used to select subjects on whom the rest of the data can be collected to learn the model.

We also test our algorithm on the well-studied PIMA Indians Diabetes data to demonstrate generality. Table 2 shows the details of these domains; a common characteristic across all domains is *class imbalance* where it is important that the most informative subjects are added to the training set.

**Table 2:** Data set details.

**Data set**	**# Pos**	**# Neg**	**# Features**		**# Examples**	
			**FO**	**PO**	**FO**	**PO**
PPMI	554	919	1	35	5	1,174
ADNI	76	260	6	69	10	294
Rare disease	87	174	6	63	10	198
PPD	38	115	8	33	6	147
PIMA	268	500	4	4	10	681

FO, Fully observed; PO, Partially observed. # Pos is number of positive examples, # Neg is number of negative examples, # Features (FO) is the number of features in the fully observed feature set, # Features (PO) is number of features in the partially observed feature set, # Examples (FO) is number of examples in the fully observed example set, # Examples (PO) is number of examples in the partially observed example set. Table appears in Natarajan et al. (2018).

### 4.1. Evaluation methodology

All data sets are partitioned as 80% train and 20% test. Results are averaged over 10 runs with a fixed test set. At each active learning step, we solicit 5 new data points until convergence. Friedman's (Friedman, 2001) gradient-boosting and SVM (Vapnik, 2013) were employed as the underlying classifier with the same settings across all methods. We used linear kernel for SVM. In order to convert the class scores to probability estimates, Platt scaling (Platt, 1999) is used to convert the SVM classifier score to probabilistic estimates using logistic transform. We wanted to perform evaluations on both linear and non-linear classifiers, hence the choice of Gradient boosting and Support Vector Machines with linear kernel. We employ KL-divergence, Hellinger distance, χ^2^ distance and Total Variation as our distance metric. We compare three different evaluation metrics: *recall* (to measure the clinically relevant *sensitivity*), *F1-score*, and geometric mean of sensitivity and specificity (*gmean*), that provide a reasonably robust evaluation in the presence of class imbalance. We considered AUCROC but as pointed out by Davis and Goadrich (2006), for severe class imbalanced data sets, this is not ideal and hence we settled on our metrics.

**Baselines:** In addition to the proposed AFE approach, we considered three other baselines: (1) Randomly choosing points to query which can potentially yield strong results when closer to convergence. This method is denoted as RND; (2) We also used uncertainty sampling on the partially observed example set using only the fully observed features. The top 5 instances that have the highest entropy were then queried for elicitable features and added to the training set. This is denoted as USObs; (3) In the third approach, we imputed all elicitable features using mode as the feature value; uncertainty sampling is then employed by computing the entropy on the full feature set, following which the top 5 values were chosen for querying. This baseline is denoted as USAll. Other active-learning baselines can be considered (such as min-max), but these generally tend to be prohibitively expensive in large feature spaces.

### 4.2. Results

We aim to answer the following questions:

Q1: Does AFE perform better than other alternative baselines for active feature elicitation?
Q2: How does the choice of divergence and classifier impact performance of AFE in different scenarios?
Q3:Is AFE robust to data imbalance and can be extended for semi-supervised settings?

#### 4.2.1. Performance of AFE as compared to other baselines

**Figure 2:**
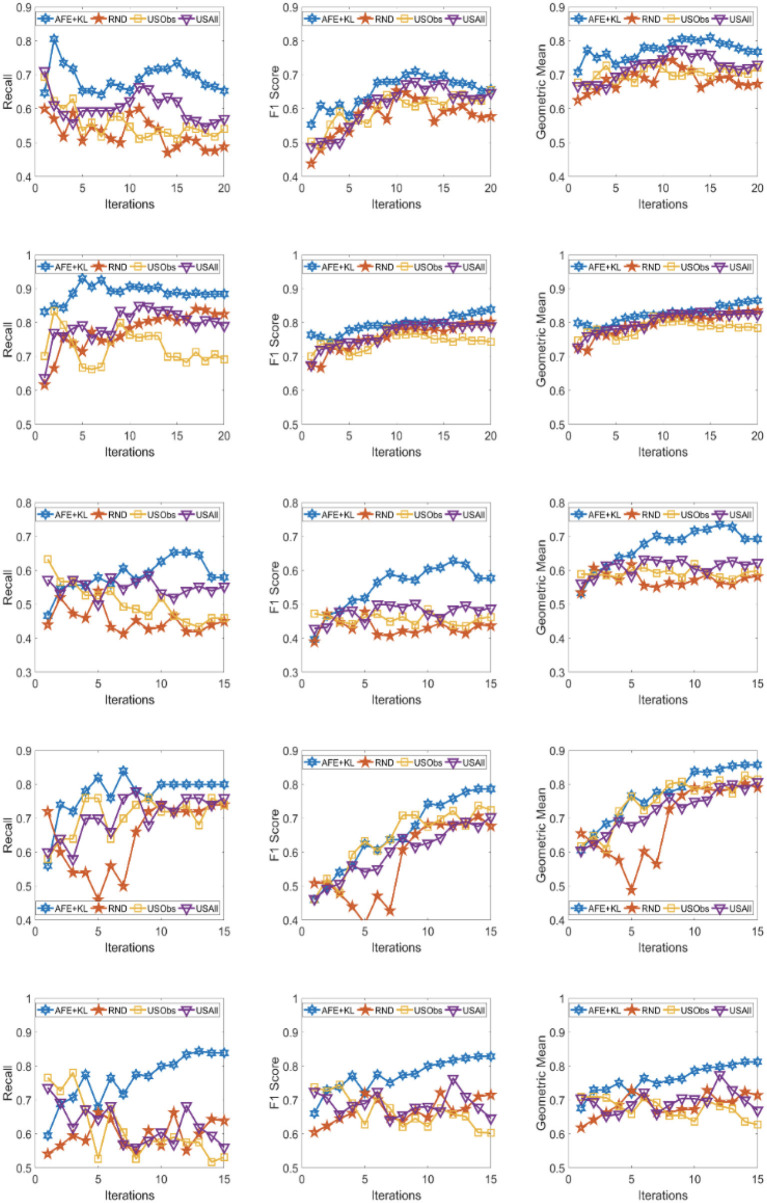
Recall **(left)**, F1 **(middle)**, g-mean **(right)** for (from top to bottom) ADNI, PPMI, Rare Disease, PPD, and PIMA. Each iteration corresponds to acquiring the 5 best examples. Classifier used is **Gradient boosting** and divergence is **KL-divergence**. Results appear in Natarajan et al. (2018).

**Figure 3:**
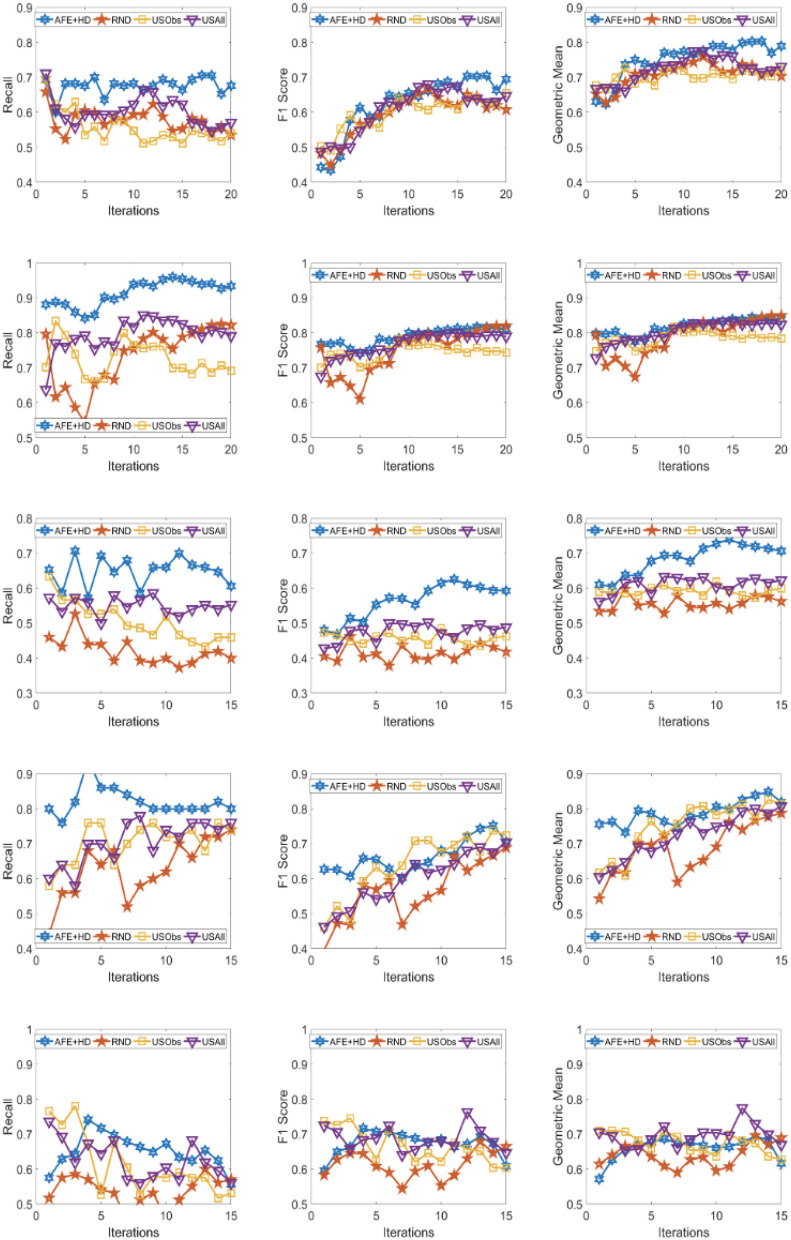
Recall **(left)**, F1 **(middle)**, g-mean **(right)** for (from top to bottom) ADNI, PPMI, Rare Disease, PPD, and PIMA. Each iteration corresponds to acquiring the 5 best examples. Classifier used is **Gradient boosting** and divergence is **Hellinger distance**.

**Figure 4:**
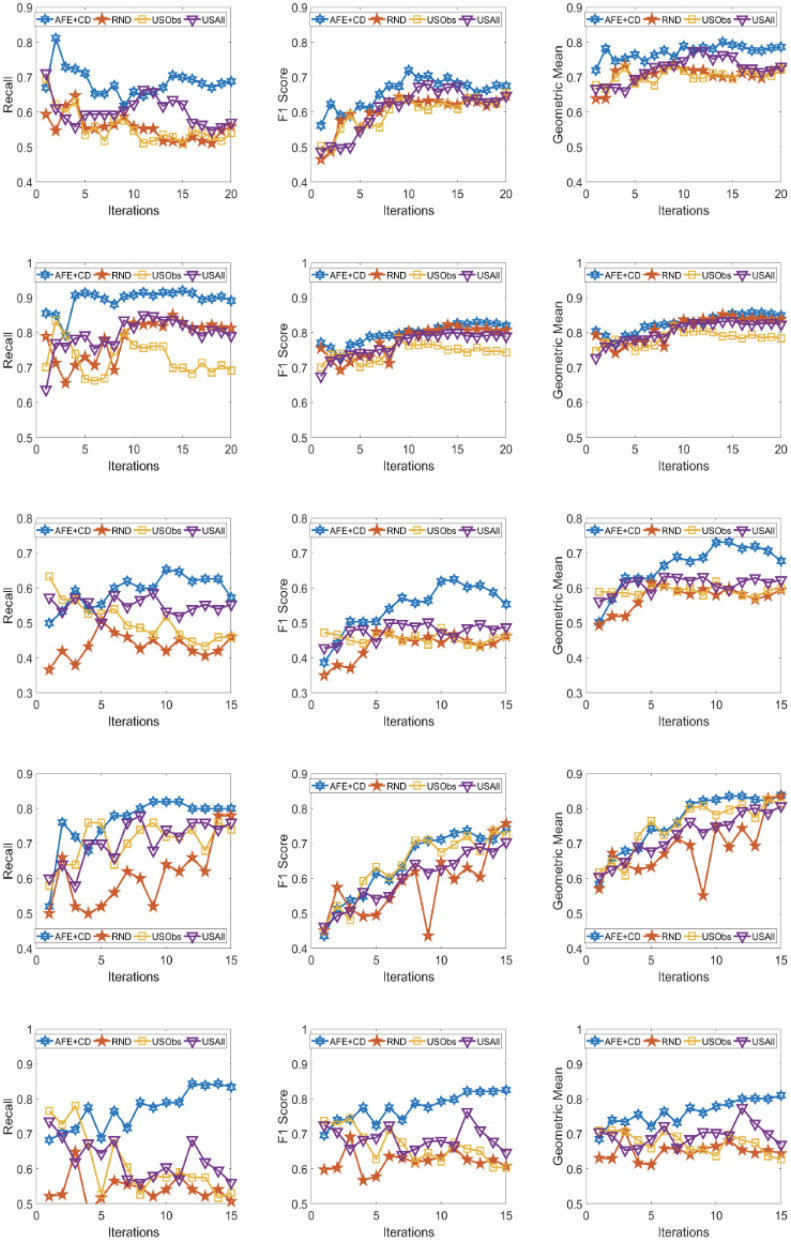
Recall **(left)**, F1 **(middle)**, g-mean **(right)** for (from top to bottom) ADNI, PPMI, Rare Disease, PPD, and PIMA. Each iteration corresponds to acquiring the 5 best examples. Classifier used is **Gradient boosting** and divergence is **χ^2^** **divergence**.

**Figure 5:**
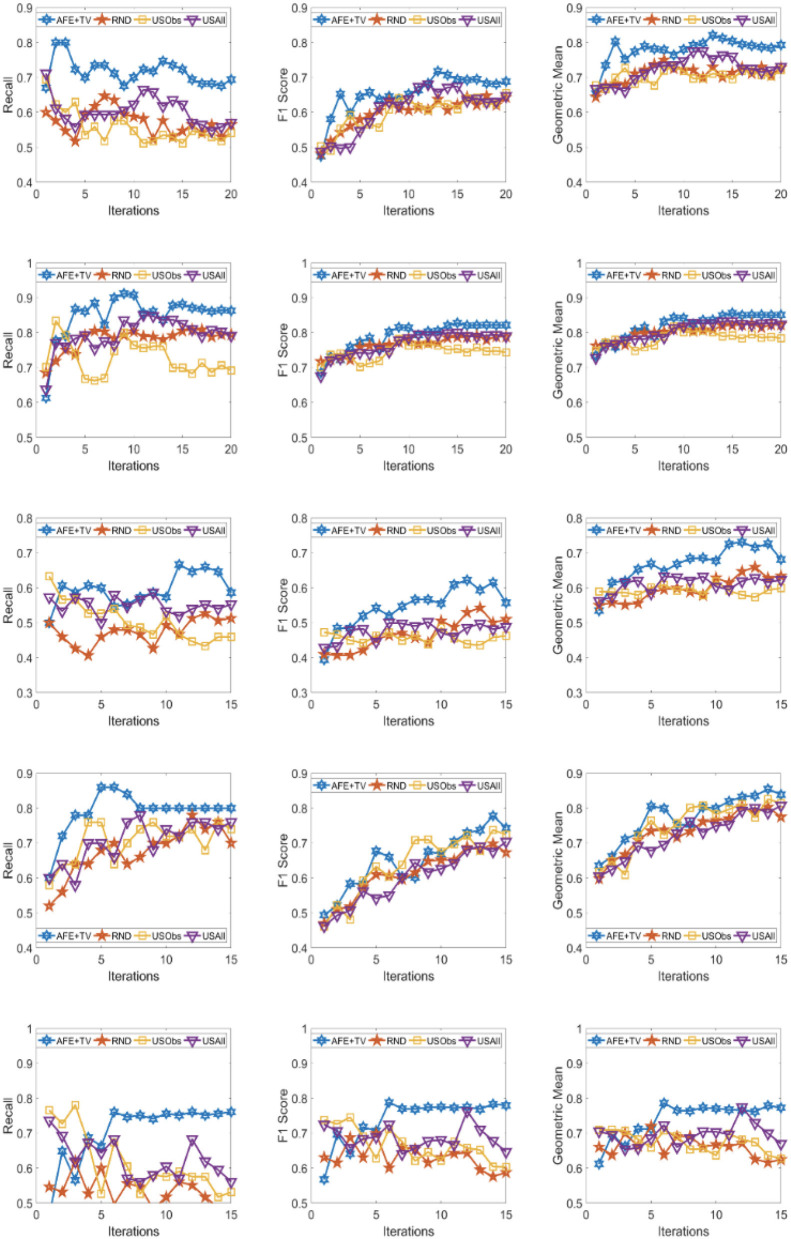
Recall **(left)**, F1 **(middle)**, g-mean **(right)** for (from top to bottom) ADNI, PPMI, Rare Disease, PPD, and PIMA. Each iteration corresponds to acquiring the 5 best examples. Classifier used is **Gradient boosting** and divergence is **Total variation**.

**Figure 6:**
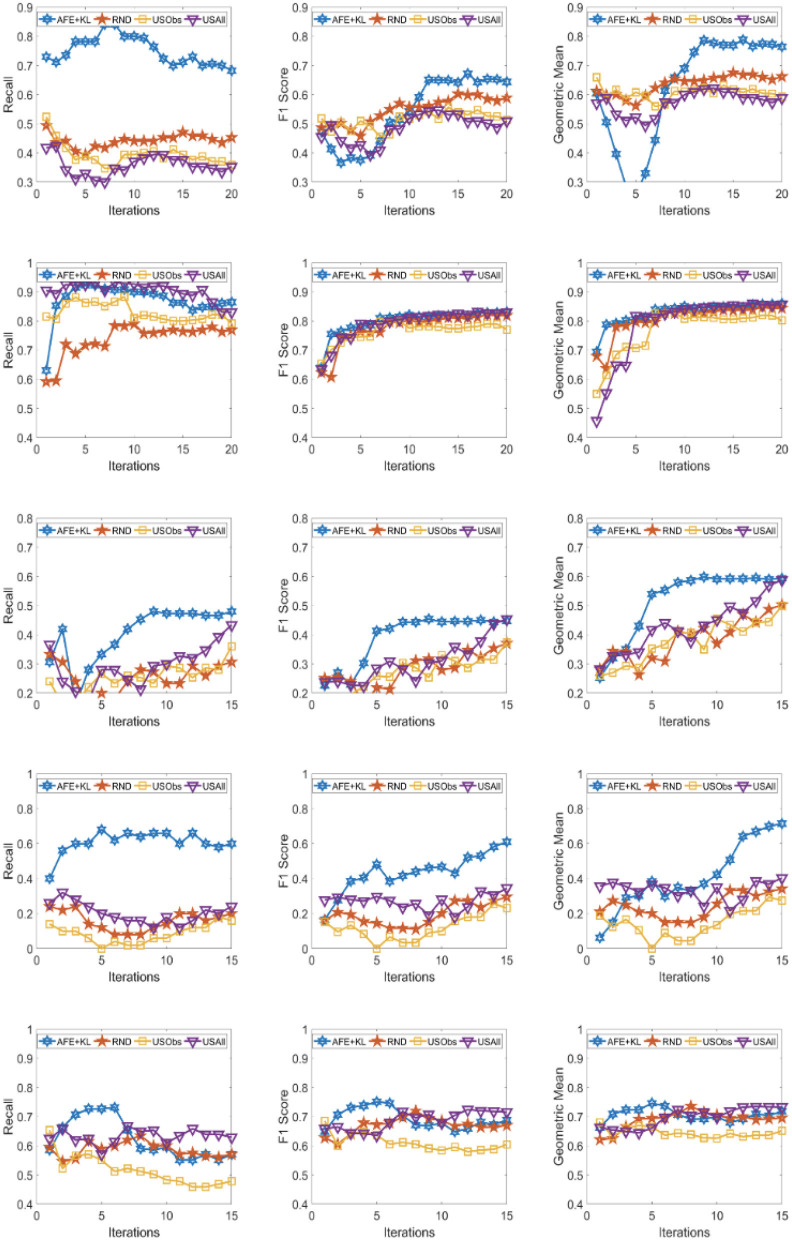
Recall **(left)**, F1 **(middle)**, g-mean **(right)** for (from top to bottom) ADNI, PPMI, Rare Disease, PPD, and PIMA. Each iteration corresponds to acquiring the 5 best examples. Classifier used is **SVM** and divergence is **KL-divergence**.

**Figure 7:**
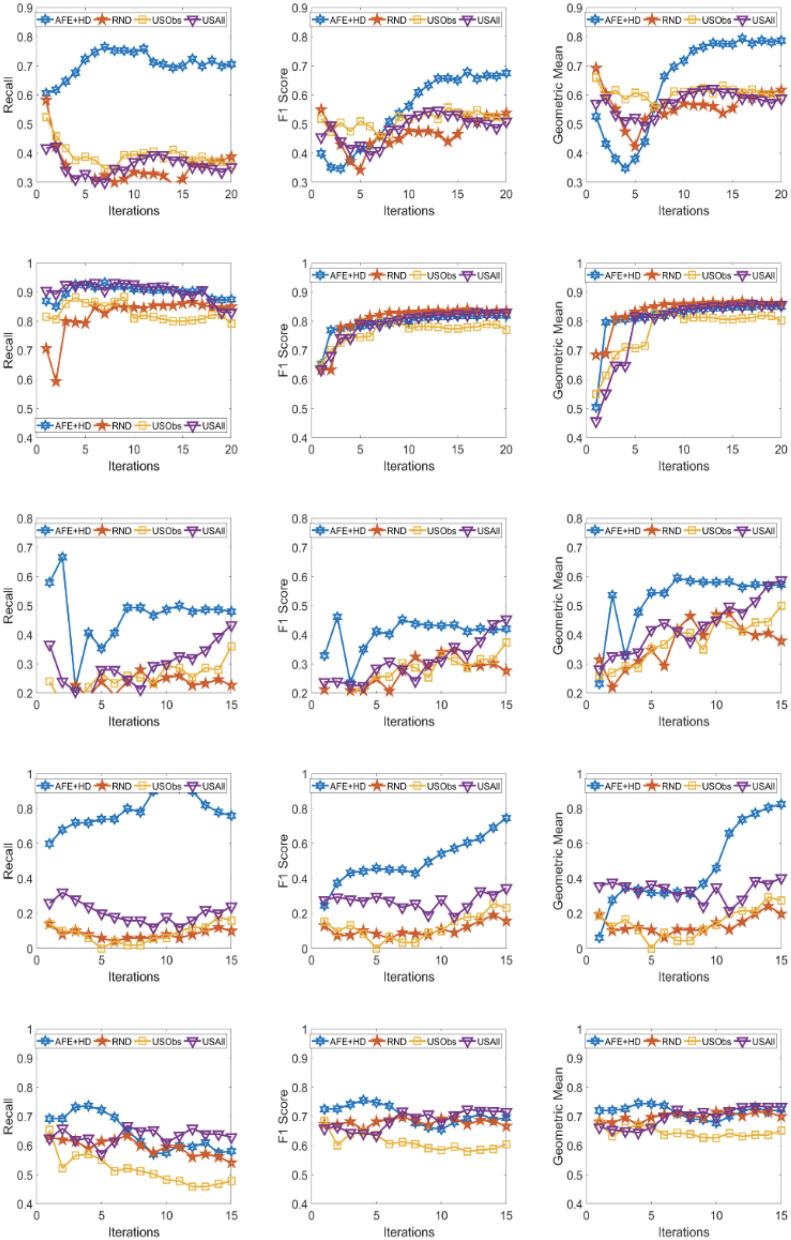
Recall **(left)**, F1 **(middle)**, g-mean **(right)** for (from top to bottom) ADNI, PPMI, Rare Disease, PPD, and PIMA. Each iteration corresponds to acquiring the 5 best examples. Classifier used is **SVM** and divergence is **Hellinger distance**.

**Figure 8:**
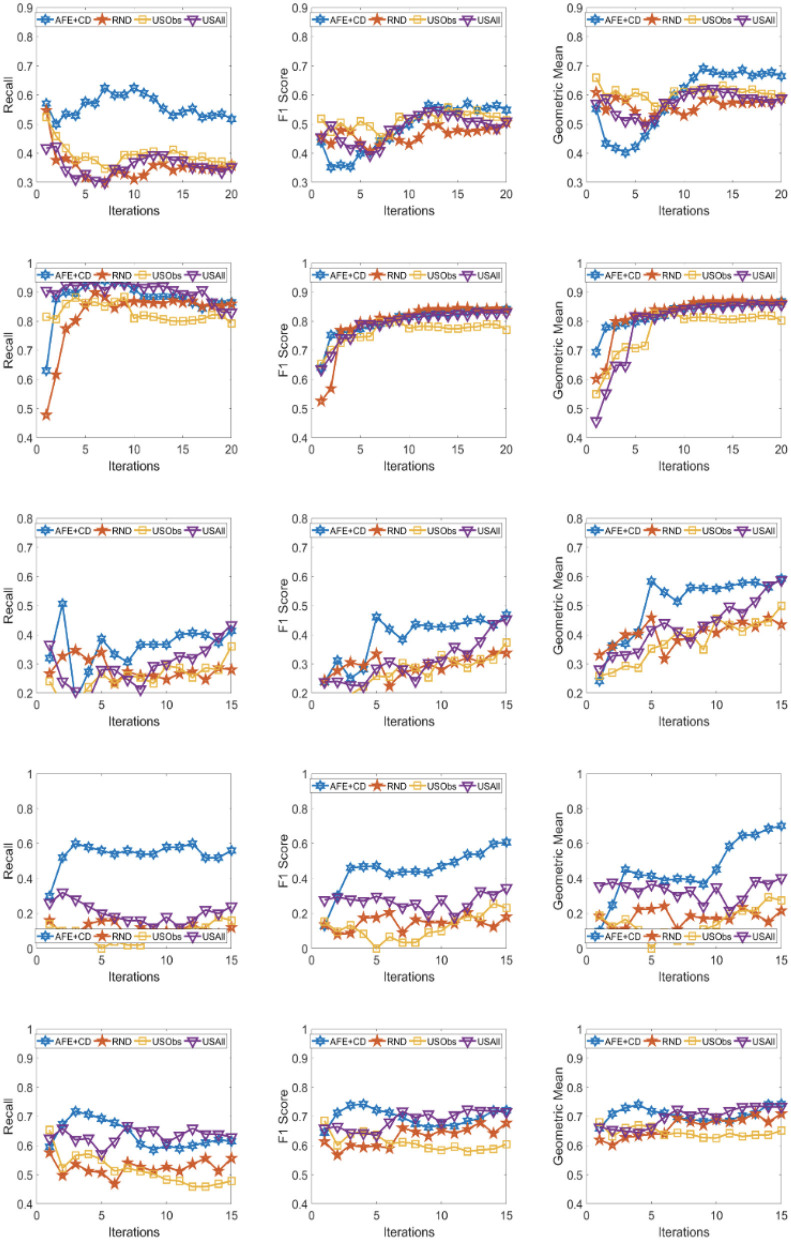
Recall **(left)**, F1 **(middle)**, g-mean **(right)** for (from top to bottom) ADNI, PPMI, Rare Disease, PPD, and PIMA. Each iteration corresponds to acquiring the 5 best examples. Classifier used is **SVM** and divergence is **χ^2^** **divergence**.

**Figure 9:**
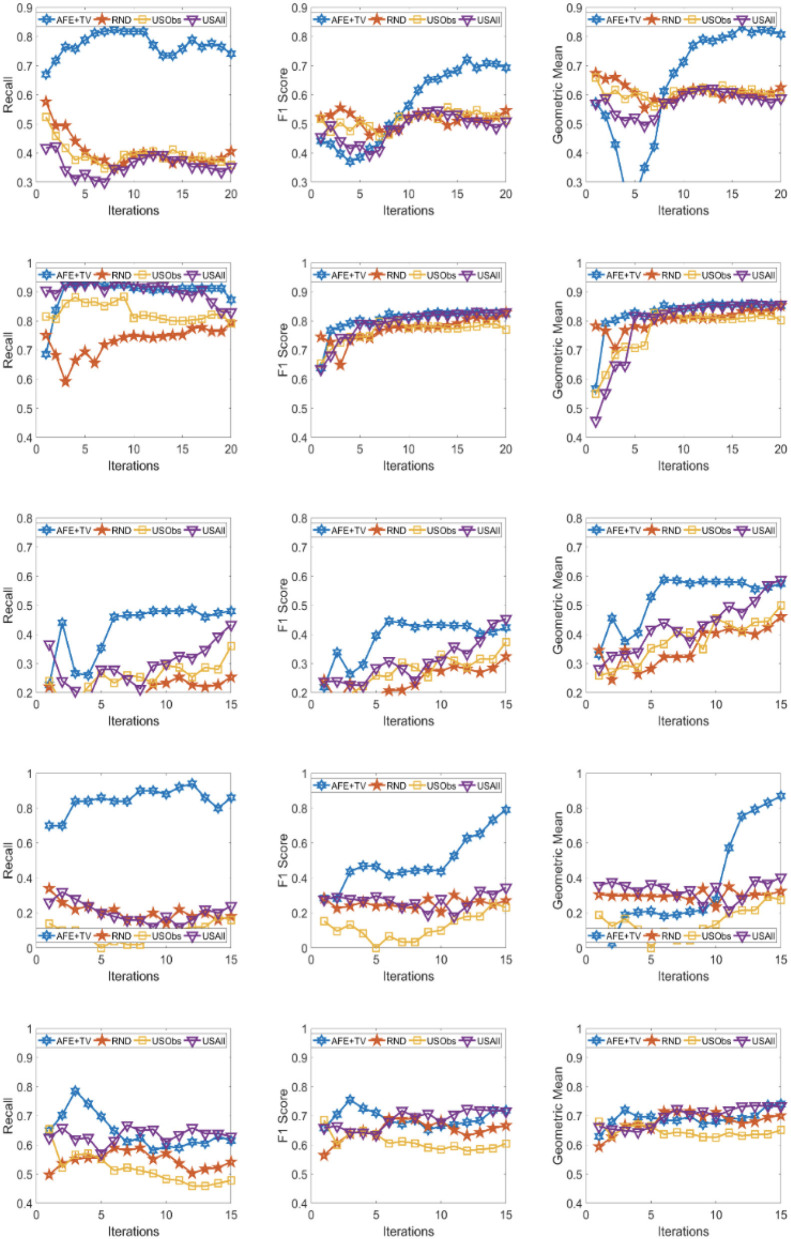
Recall **(left)**, F1 **(middle)**, g-mean **(right)** for (from top to bottom) ADNI, PPMI, Rare Disease, PPD, and PIMA. Each iteration corresponds to acquiring the 5 best examples. Classifier used is **SVM** and divergence is **Total variation**.

The results across the five domains and all the three metrics are presented for Gradient Boosting (GB) and SVM Classifier (linear kernel) with the different distance metric in Figures 2–9. The various distance metric considered for the experiments are KL-divergence, Hellinger distance, χ^2^ divergence, and Total-variation. Specifically, the experimental results of AFE with GB & KL-divergence, Hellinger distance, χ^2^ divergence and Total-variation are shown in Figures 2–5, respectively and the results of AFE with SVM & KL-divergence, Hellinger distance, χ^2^ divergence and Total-variation are shown in Figures 6–9, respectively. It can be seen from the above mentioned figures that AFE clearly outperforms the other baselines in Recall, F1-score, and gmean and converges faster than the other baselines. These results on the real-world data sets clearly demonstrates that AFE is a general framework where the classifier and appropriate divergence metric can be plugged in according to the domain, data, prior knowledge of the conditional distributions as well as prior knowledge of the available and elicitable feature subsets.

It can be observed that AFE outperforms the random baseline RND on all domains across all metrics for all combinations of classifier and distance metric, specifically in recall in the first few iterations in 4 out of the 5 domains as seen in Figures 2–9 where the effect of choosing the most informative set of examples can have the maximal impact on the classifier performance. As expected, the variance in recall due to random selection of examples is high. This can be seen in the leftmost column of all the figures, where the performance does not increase steadily in all domains. Similar observations can be made for the geometric mean and F1-score (middle and right-most column of all the figures).

Observe that as we add more informative examples, the performance improves significantly for AFE over the rest of the baselines. This demonstrates that the gains are not necessarily in the beginning alone. Adding more useful examples can construct a more robust training set that can lead the classifier to superior performance. One of the reasons that we are averaging over 10 runs is to alleviate/minimize the effect of sampling bias in the construction of training set (particularly in the initial samples). As expected, the variance is initially on the higher side indicating the effect of sampling bias on smaller training sets but it decreases as more examples are added. However, this effect is minimal for AFE that chooses good training examples compared to other methods. Understanding the effect of the initial choice of samples on the performance of the classifier is itself an interesting future research direction.

Similar results can be observed when comparing AFE to USObs and USAll in that AFE consistently outperforms the two active learning baselines across all the domains in all the metrics as seen in Figures 2–9. We hypothesize that the use of better imputation techniques may improve performance of USAll. The difference between AFE and USAll in recall and gmean across all domains is statistically significant in several iterations. This suggests that other imputation techniques may marginally improve the performance, but may not influence the final performance and a good selection of examples is necessary. The experimental results helps answer **Q1** affirmatively.

Another natural question to ask is how the variance in performance of the different methods tend to behave across all data sets? It was generally observed that the AFE had the smallest variance both in the selection of the first few examples and in the last few iterations of the algorithms. The variance of AFE across all data sets was at least half the variance of the Random baseline (RND) on an average in the first few iterations and as low as 10% of random selection's variance in later iterations. This is also consistent across all metrics for all the combinations of classifier and divergence metric. When compared to USObs and USAll, in general, the average variance of AFE was lower across all metrics and all data sets. While AFE's variance is significantly better than the random selection, the differences to the uncertainty methods, while better, are not necessarily significant.

#### 4.2.2. Impact of the choice of divergence and classifier on performance of AFE

AFE certainly performs better on certain data sets with a particular combination of classifier and divergence metric. We compared the Recall, F1 score and gmean achieved in the final iteration of AFE to see if certain divergence metric helps the final training model to perform better. For the ADNI data set, **Total variation** performs the best when compared against other divergence metrics for both the classifiers in Recall and gmean for the final training iteration (Figures 5, 9). For the PPMI data set, **Hellinger distance** emerges the winner for both the classifiers in Recall as per Figures 3, 7. When compared between the 2 classifiers, **Gradient Boosting** is always ranked higher than SVM for the PPMI and PPD data set across all the divergence metrics except Total-variation on Recall. Total variation along with SVM works better on these data sets for Recall as seen in Figure 9. These show that AFE certainly performs better with some classifier and divergence metric setting depending on the data set and how much the elicitable feature subsets in the data set helps in predicting the target variable.

**Figure 10:**
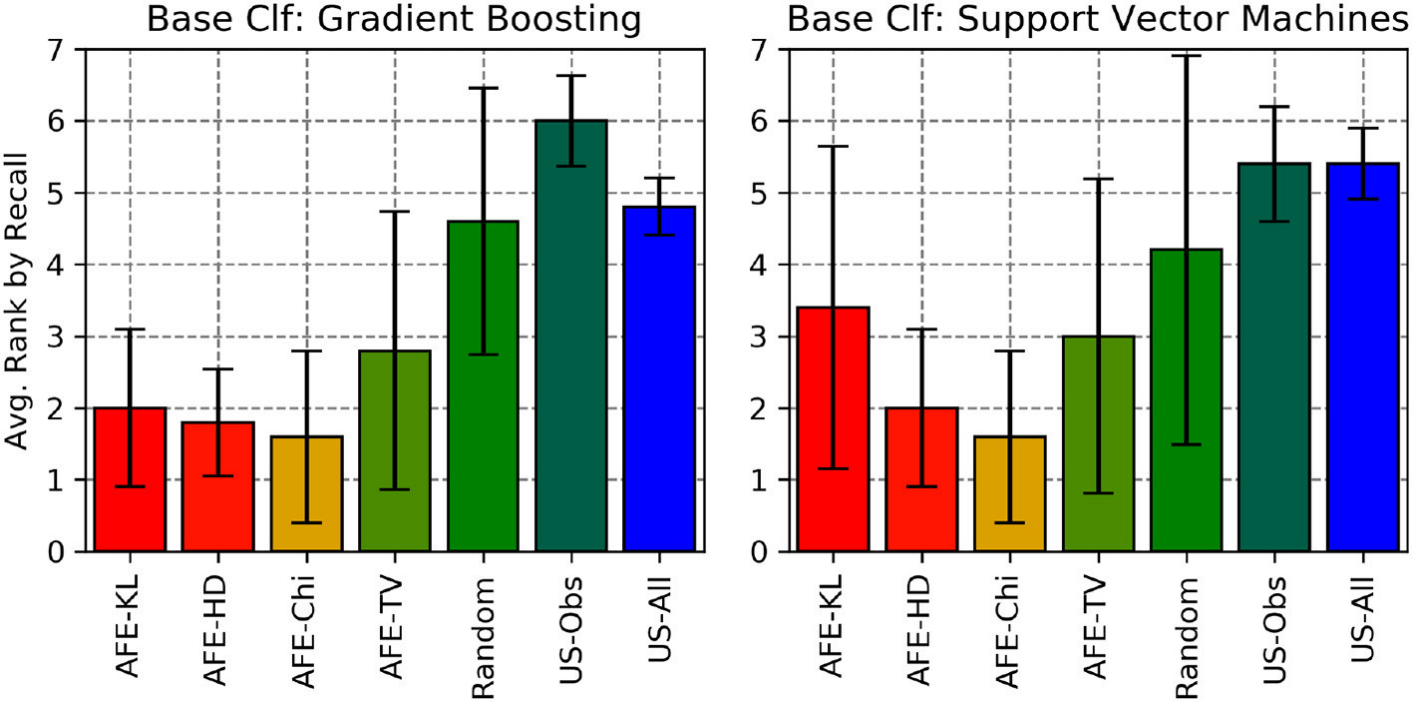
Average rankings of Recall for all the algorithms considered across all the data sets.

**Figure 11:**
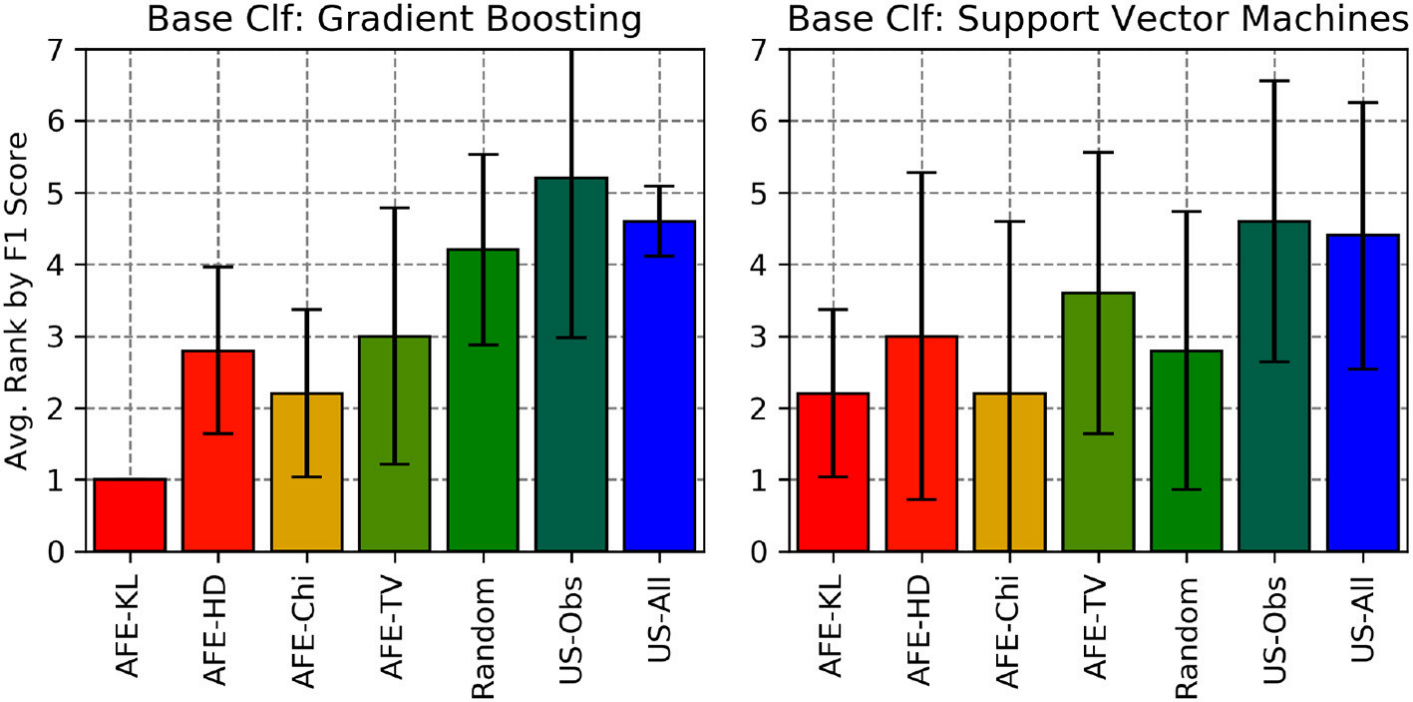
Average rankings of F1 for all the algorithms considered across all the data sets.

**Figure 12:**
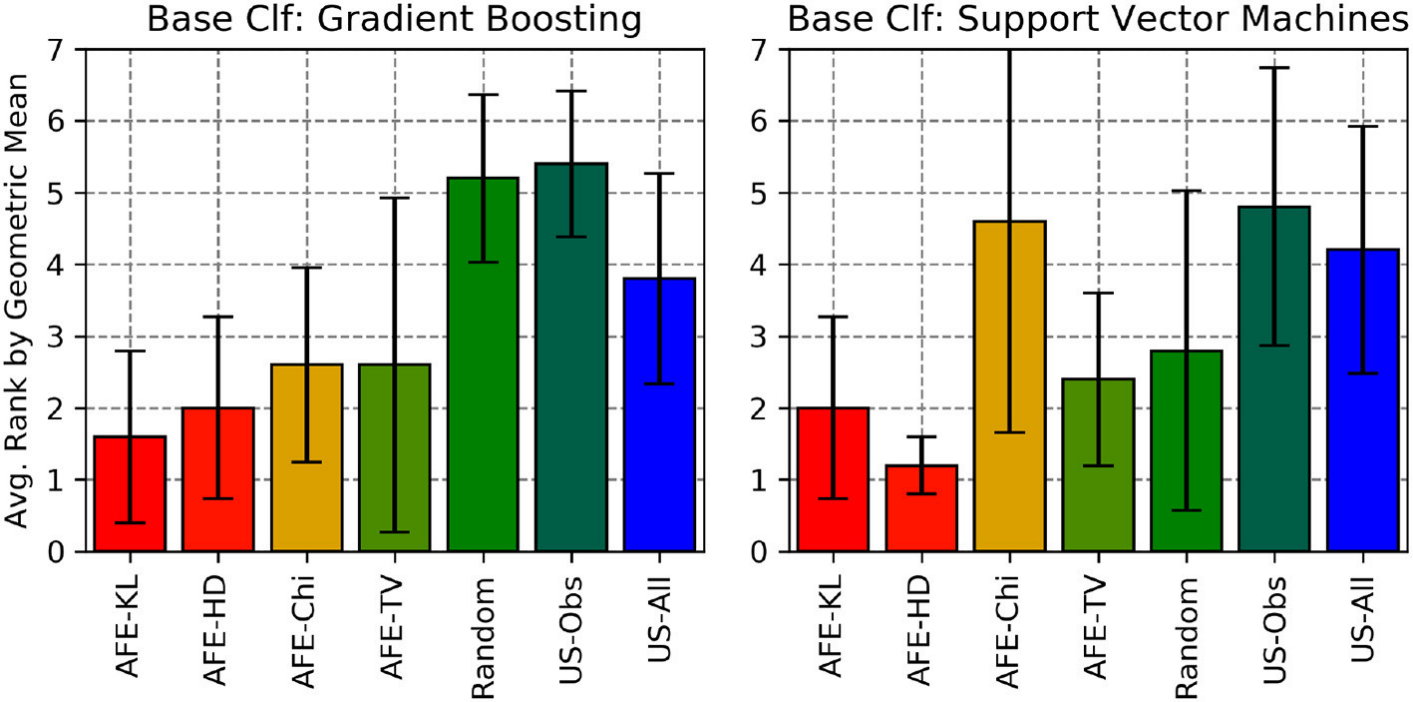
Average rankings of gmean for all the algorithms considered across all the data sets.

We also calculated the average rank by Recall, F1-score and gmean of the various divergence metric with AFE across all the 5 data sets (for SVM and Gradient Boosting) and compared them against the 3 baselines (Random, US-Obs and US-All) as shown in Figures 10–12, respectively. Here, two (or more) algorithms are ranked equally if they are within 3% of each other's performance in that metric. From these plots, AFE with various divergence metric emerges as the clear winner than the other 3 baselines. For Gradient Boosting classifier, the average ranking by F1 score and geometric mean suggests that AFE+KL is better than any other combination of AFE with other divergence metrics. When comparing the average rank of F1-score between Gradient Boosting and SVM from Figure 11, it can be seen that AFE with KL divergence for Gradient Boosting has the lowest average rank than any other combination of classifier and divergence metric. Since F1-score is a function of recall and precision, we can say that AFE+KL with Gradient Boosting on an average is better than any other combination across all the data sets. From the above experimental results, we conclude that no particular combination of classifier and divergence metrics stands the best; the combination is dependent on the domain and data distribution.

#### 4.2.3. Robustness of AFE to class imbalance

As shown in Table 2, all domains are imbalanced. For PPMI and PPD domains where the class prior is skewed, it can be seen clearly that the AFE achieves a recall of over 0.8 after just a few early iterations for Gradient Boosting classifier as seen in Figures 2–5. This demonstrates that AFE can identify the most important examples that allow for increasing the clinically relevant sensitivity effectively enabling us to answer **Q3** affirmatively. Our results in all domains show that this method achieves high recall without significantly sacrificing precision making it an ideal choice for semi-supervised imbalanced data sets. The intuition here is that because AFE obtains high recall across all data sets, in domains where many examples are labeled, it provides an opportunity for selecting the right sets of examples that can be labeled or extended with more features. Although the proposed approach is not a semi-supervised one, it provides an opportunity for developing methods that can learn from partially labeled data.

AFE starts with a very small training sample and iteratively acquires the data points which are farther from the conditional distribution of the target given all the features *P*(*Y*|**X**_*o*_, **X**_*u*_) thus helping in approximating this distribution better with every iteration. Experimentally, we have seen that AFE in general selects data points to maintain the class balance in the training set. This is because, whenever, samples from a certain class dominates the training set, the conditional distribution of data points with minority class deviates from *P*(*Y*|**X**_*o*_, **X**_*u*_) and hence, these data points are acquired by AFE which can aid in sampling for imbalanced data sets. From the above discussion, we conclude that AFE is effective in handling imbalanced data and can be easily extended to semi-supervised settings.

Our original motivation was to identify the set of subjects on whom to perform specific lab tests, given some basic information about the potential recruits. Our algorithm does not use any extra information about the potential recruits beyond the observed features and their labels (whether they are cases or controls) for identifying the best set of subjects to elicit more information about. Secondly, we do not make any assumptions about the underlying distributions of the data or the classifier employed while learning. We make effective use of the fully observed data (from possibly a related study) by using them to compute the distance with the potential cohorts. Finally, AFE is able to perform well on data with class imbalance while balancing the cost of eliciting features. This demonstrates that AFE is indeed faithful to the original goals and is effective in modeling clinical data as shown in our experiments on several real world data sets.

AFE starts with a very small training sample and iteratively acquires the data points which are farther from the conditional distribution of the target given all the features *P*(*Y*|**X**_*o*_, **X**_*u*_) thus helping in approximating this distribution better with every iteration. Experimentally, we have seen that AFE in general picks data points to maintain the class balance in the training set. This is because, whenever, samples from a certain class dominates the training set, the conditional distribution of data points with minority class deviates from *P*(*Y*|**X**_*o*_, **X**_*u*_) and hence, these data points are acquired by AFE which can aid in sampling for imbalanced data sets thus answering **Q9** affirmatively.

## 5. Conclusion

We considered the problem of eliciting new sets of features based on a small amount of fully observed data. We address this problem specifically in the context of medical domains with severe class imbalance. Our proposed approach and problem setting has practical applications in health care domains especially in context to participant recruitment for a clinical/smart device study where easily available features can be collected for everyone and potential participants for whom expensive features needs to be collected can be identified in order to build a budget friendly machine learning model. Our proposed active feature elicitation approach computes the similarity between the potentially interesting examples with the fully observed examples and chooses the most significantly different examples to elicit the feature information. These are then added to the full observed set and the process continues until convergence. Experiments on four real high-impact medical tasks demonstrate the effectiveness and efficiency of the proposed approach. Our approach has a few salient features—it is domain, model, and distance, i.e., representation agnostic in that any reasonable classifier and a compatible distance metric for a specific domain can be employed in a plug and play manner.

We currently elicit all elicitable features for every chosen example at every iteration. One could extend this work by identifying sets of features that are most informative for every example (i.e., the most relevant lab test for each subject) along the lines of the work of Krishnapuram et al. (2005) that addressed a similar problem using multi-view, co-training setting which could allow for the realization of the vision of personalized medicine. Another interesting future research direction could be to identify groups of examples (sub-populations) that would provide the most information at each iteration. Extending the work to handle fully relational/graph data is another possible direction. Finally, rigorous evaluation of the approach on more clinical data sets can yield more interesting insights into the proposed approach.

## Data availability statement

The datasets analyzed for this study can be found in the following repository: PIMA-https://github.com/sridas123/Active-Feature-Elicitation/tree/master/Data/Pima. The other real-world medical data sets are not publicly available due to ethical considerations.

## Author contributions

SN and PR contributed equally to the ideation. SD led the algorithmic development and experimental evaluations. NR contributed to experimental evaluations. GK contributed to algorithmic development. NR, PR, and GK contributed to the manuscript preparation which was led by SN and SD. All authors contributed to the article and approved the submitted version.

## Footnotes

^1^ www.loni.ucla.edu/ADNI

^2^An earlier version of the paper had appeared in IJCAI 2018 (Natarajan et al., 2018). We have significantly expanded on the paper by including additional theoretical insights and experiments using different divergence measures and classifiers.

^3^Genome sequencing would best exemplify such a scenario.

^4^See Deza and Deza, 2013 for other useful distance functions.

^5^ http://prefrontal.org/blog/2008/05/brain-art-aal-patchwork

^6^ http://www.slicer.org

## References

[B1] Bilgic M. Getoor L. (2007). VOILA: efficient feature-value acquisition for classification, in AAAI'07: Proceedings of the 22nd National Conference on Artificial intelligence (Vancouver, CA), 1225–1230.

[B2] Boutilier C. Regan K. Viappiani P. (2009). Online feature elicitation in interactive optimization, in ICML '09: Proceedings of the 26th Annual International Conference on Machine Learning (Montreal, QC), 73–80.

[B3] Chai X. Deng L. Yang Q. Ling C. X. (2004). Test-cost sensitive naive bayes classification, in ICDM (Brighton), 51–58.

[B4] Cichocki A. Amari S. (2010). Families of alpha- beta- and gamma- divergences: flexible and robust measures of similarities. Entropy 12, 1532–1568. 10.3390/e12061532

[B5] Cieslak D. A. Hoens T. R. Chawla N. V. Kegelmeyer W. P. (2012). Hellinger distance decision trees are robust and skew-insensitive. Data Mining Knowl. Discov. 24, 136–158. 10.1007/s10618-011-0222-1

[B6] Csiszár I. (1967). Information measures of difference of probability distributions and indirect observations. Studia Sci. Math. Hungar. 2, 299–318.

[B7] Das S. Iyer R. Natarajan S. (2021). A clustering based selection framework for cost aware and test-time feature elicitation, in 8th ACM IKDD CODS and 26th COMAD (Bangalore), 20–28.

[B8] Davis J. Goadrich M. (2006). The relationship between precision-recall and ROC curves, in ICML (Pittsburgh, PA), 233–240.

[B9] Deza M. M. Deza E. (2013). Encyclopedia of Distances. Springer.

[B10] Druck G. Settles B. McCallum A. (2009). Active learning by labeling features, in EMNLP (Singapore), 81–90.

[B11] Dulac-Arnold G. Denoyer L. Preux P. Gallinari P. (2011). Datum-wise classification: a sequential approach to sparsity, in ECML PKDD (Athens), 375–390.

[B12] Dwork C. Hardt M. Pitassi T. Reingold O. Zemel R. (2012). Fairness through awareness, in Proceedings of the 3rd Innovations in Theoretical Computer Science Conference (Cambridge, MA).

[B13] Friedman J. H. (2001). Greedy function approximation: a gradient boosting machine. Ann. Stat. 29, 1189–1232. 10.1214/aos/1013203451

[B14] Gillen S. Jung C. Kearns M. Roth A. (2018). Online learning with an unknown fairness metric, in NIPS (Montreal, QC).

[B15] Gong W. Tschiatschek S. Nowozin S. Turner R. E. Hernández-Lobato J. M. Zhang C. (2019). Icebreaker: element-wise efficient information acquisition with a bayesian deep latent Gaussian model, in Advances in Neural Information Processing Systems (Vancouver, BC), 14791–14802.

[B16] He H. Eisner J. Daume H. (2012). Imitation learning by coaching, in NIPS, 3149–3157.

[B17] Hofmann T. Buhmann J. M. (1998). Active data clustering, in NIPS (Denver, CO), 528–534.

[B18] Huang S.-J. Xu M. Xie M.-K. Sugiyama M. Niu G. Chen S. (2018). Active feature acquisition with supervised matrix completion, in KDD (London), 1571–1579.

[B19] Janisch J. Pevnỳ T. Lisỳ V. (2019). Classification with costly features using deep reinforcement learning, in AAAI (Honolulu, HI).

[B20] Judah K. Fern A. Tadepalli P. Goetschalckx R. (2014). Imitation learning with demonstrations and shaping rewards, in AAAI (Quebec City, QC), 1890–1896.

[B21] Kanani P. Melville P. (2008). Prediction-time active feature-value acquisition for cost-effective customer targeting, in Workshop on Cost Sensitive Learning at NIPS.

[B22] Kanani P. H. McCallum A. Pal C. (2007). Improving author coreference by resource-bounded information gathering from the web, in IJCAI, 429–434.

[B23] Kedem D. Tyree S. Sha F. Lanckriet G. Weinberger K. Q. (2012). Non-linear metric learning, in Advances in Neural Information Processing Systems 25 (NIPS 2012) (Lake Tahoe), 2582–2590.

[B24] Kleinberg J. Mullainathan S. Raghavan M. (2017). Inherent trade-offs in the fair determination of risk scores, in ACM Conference on Innovations in Theoretical Computer Science.14698953

[B25] Krause A. Guestrin C. (2009). Optimal value of information in graphical models. J. Artif. Intell. Res. 35, 557–591. 10.1613/jair.2737

[B26] Krishnapuram B. Williams D. Xue Y. Carin L. (2005). Active learning of features and labels, in Workshop on Learning with Multiple Views at ICML.

[B27] Kunapuli G. Shavlik J. (2012). Mirror descent for metric learning: a unified approach, in ECML PKDD (Bristol), 859–874.

[B28] Lewis D. D. Catlett J. (1994). Heterogeneous uncertainty sampling for supervised learning, in ICML (New Brunswick, NJ), 148–156.

[B29] Lewis D. D. Gale W. A. (1994). A sequential algorithm for training text classifiers, in SIGIR (Dublin), 3–12.

[B30] Ling C. X. Yang Q. Wang J. Zhang S. (2004). Decision trees with minimal costs, in ICML (Banff), 69.

[B31] Lizotte D. J. Madani O. Greiner R. (2003). Budgeted learning of naive-bayes classifiers, in UAI'03: Proceedings of the Nineteenth Conference on Uncertainty in Artificial Intelligence (Acapulco), 378–385.

[B32] Lopes M. Melo F. Montesano L. (2009). Active learning for reward estimation in inverse reinforcement learning, in ECML PKDD (Bled), 31–46.

[B33] MacLeod H. Yang S. Oakes K. Connelly K. Natarajan S. (2016). Identifying rare diseases from behavioural data: a machine learning approach, in 2016 IEEE First International Conference on Connected Health: Applications, Systems and Engineering Technologies (CHASE) (Washington, DC), 130–139.

[B34] Marek K. Jennings D. (2011). The Parkinson Progression Marker Initiative (PPMI). Prog. Neurobiol. 95, 629–635. 10.1016/j.pneurobio.2011.09.005 21930184 PMC9014725

[B35] Melville P. Saar-Tsechansky M. Provost F. Mooney R. (2004). Active feature-value acquisition for classifier induction, in Fourth IEEE International Conference on Data Mining (ICDM'04) (Brighton), 483–486.

[B36] Melville P. Saar-Tsechansky M. Provost F. Mooney R. (2005). An expected utility approach to active feature-value acquisition, in Fifth IEEE International Conference on Data Mining (ICDM'05) (Houston, TX), 745–748.

[B37] Nan F. Saligrama V. (2017). Adaptive classification for prediction under a budget, in Advances in Neural Information Processing Systems 30 (NIPS 2017) (Long Beach, CA).

[B38] Nan F. Wang J. Saligrama V. (2015). Feature-budgeted random forest, in ICML'15: Proceedings of the 32nd International Conference on International Conference on Machine Learning (Lille).

[B39] Nan F. Wang J. Saligrama V. (2016). Pruning random forests for prediction on a budget, in NIPS'16: Proceedings of the 30th International Conference on Neural Information Processing Systems (Barcelona).

[B40] Natarajan S. Das S. Ramanan N. Kunapuli G. Radivojac P. (2018). On whom should I perform this lab test next? An active feature elicitation approach, in Proceedings of the Twenty-Seventh International Joint Conference on Artificial Intelligence (Stockholm), 3498–3505.

[B41] Natarajan S. Prabhakar A. Ramanan N. Bagilone A. Siek K. Connelly K. (2017). Boosting for postpartum depression prediction, in 2017 IEEE/ACM International Conference on Connected Health: Applications, Systems and Engineering Technologies (CHASE) (Philadelphia, PA), 232–240.

[B42] Odom P. Natarajan S. (2016). Active advice seeking for inverse reinforcement learning, in AAMAS '16: Proceedings of the 2016 International Conference on Autonomous Agents & Multiagent Systems (Singapore), 512–520.

[B43] Platt J. (1999). Probabilistic outputs for support vector machines and comparisons to regularized likelihood methods, in Advances in Large Margin Classifiers, 61–74.

[B44] Raghavan H. Madani O. Jones R. (2006). Active learning with feedback on features and instances. J. Mach. Learn. Res. 7, 1655–1686.30740605

[B45] Saar-Tsechansky M. Melville P. Provost F. (2009). Active feature-value acquisition. Manag. Sci. 55, 664–684. 10.1287/mnsc.1080.0952

[B46] Settles B. (2012). Active learning, in Synthesis Lectures on Artificial Intelligence and Machine Learning (Morgan & Claypool Publishers), 6. 10.2200/S00429ED1V01Y201207AIM018

[B47] Shim H. Hwang S. J. Yang E. (2018). Joint active feature acquisition and classification with variable-size set encoding, in NIPS (Montreal, QC), 1368–1378.

[B48] Smith J. W. Everhart J. E. Dickson W. C. Knowler W. C. (1988). Using the ADAP learning algorithm to forecast the onset of diabetes mellitus, in Proc Annu Symp Comput Appl Med Care, 261–265.

[B49] Thahir M. Sharma T. Ganapathiraju M. K. (2012). An efficient heuristic method for active feature acquisition and its application to protein-protein interaction prediction. BMC Proc. 6(Suppl 7), S2. 23173746 10.1186/1753-6561-6-S7-S2 PMC3504800

[B50] Tong S. Koller D. (2000). Active learning for parameter estimation in Bayesian networks, in NIPS (Denver, CO), 647–653.

[B51] Tong S. Koller D. (2001a). Active learning for structure in Bayesian networks, in IJCAI (Seattle, WA), 863–869.

[B52] Tong S. Koller D. (2001b). Support vector machine active learning with applications to text classification. J. Mach. Learn. Res. 2, 45–66. 10.1162/153244302760185243

[B53] Vapnik V. (2013). The Nature of Statistical Learning Theory. Springer Science & Business Media.

[B54] Wang J. Trapeznikov K. Saligrama V. (2015). Efficient learning by directed acyclic graph for resource constrained prediction, in NIPS (Montreal, QC).

[B55] Xu Z. Kusner M. Weinberger K. Chen M. (2013). Cost-sensitive tree of classifiers, in ICML (Atlanta, GA).

[B56] Xu Z. Weinberger K. Q. Chapelle O. (2012). The greedy miser: learning under test-time budgets, in ICML (Edinburgh).

[B57] Zheng Z. Padmanabhan B. (2002). On active learning for data acquisition, in ICDM (Maebashi City), 562–569.

